# Whole Genome Sequencing and Re-sequencing of the Sable Antelope (*Hippotragus niger*): A Resource for Monitoring Diversity in *ex Situ* and *in Situ* Populations

**DOI:** 10.1534/g3.119.400084

**Published:** 2019-04-18

**Authors:** Klaus-Peter Koepfli, Gaik Tamazian, David Wildt, Pavel Dobrynin, Changhoon Kim, Paul B. Frandsen, Raquel Godinho, Andrey A. Yurchenko, Aleksey Komissarov, Ksenia Krasheninnikova, Sergei Kliver, Sofia Kolchanova, Margarida Gonçalves, Miguel Carneiro, Pedro Vaz Pinto, Nuno Ferrand, Jesús E. Maldonado, Gina M. Ferrie, Leona Chemnick, Oliver A. Ryder, Warren E. Johnson, Pierre Comizzoli, Stephen J. O’Brien, Budhan S. Pukazhenthi

**Affiliations:** * Smithsonian Conservation Biology Institute, Center for Species Survival, National Zoological Park, Front Royal, VA, 22630 and Washington, DC 20008; † Theodosius Dobzhansky Center for Genome Bioinformatics, Saint Petersburg State University, St. Petersburg 199034, Russia; ‡ Macrogen Inc., Seoul 08511, Korea; § Department of Plant and Wildlife Sciences, Brigham Young University, Provo, UT, 84602; ** CIBIO/InBIO - Centro de Investigacão em Biodiversidade e Recursos Genéticos, Universidade do Porto, Campus Agrário de Vairão, 4485-661 Vairão, Portugal; †† Departamento de Biologia, Faculdade de Ciências, Universidade do Porto, Rua do Campo Alegre s/n, 4169-007 Porto, Portugal; ‡‡ Department of Zoology, University of Johannesburg, Auckland Park 2006, South Africa; §§ Smithsonian Conservation Biology Institute, Center for Conservation Genomics, National Zoological Park, Washington, DC 20008; *** Disney’s Animal Kingdom, Animals, Science and Environment, Lake Buena Vista, FL 32830; ††† San Diego Zoo Institute for Conservation Research, Escondido, CA 92027; ‡‡‡ Walter Reed Biosystematics Unit, Museum Support Center, Smithsonian Institution, Suitland, MD 20746; §§§ Guy Harvey Oceanographic Center, Nova Southeastern University, Ft Lauderdale, FL 33004

**Keywords:** *Hippotragus niger*, sable antelope, genome assembly, conservation genetics, Bovidae

## Abstract

Genome-wide assessment of genetic diversity has the potential to increase the ability to understand admixture, inbreeding, kinship and erosion of genetic diversity affecting both captive (*ex situ*) and wild (*in situ*) populations of threatened species. The sable antelope (*Hippotragus niger*), native to the savannah woodlands of sub-Saharan Africa, is a species that is being managed *ex situ* in both public (zoo) and private (ranch) collections in the United States. Our objective was to develop whole genome sequence resources that will serve as a foundation for characterizing the genetic status of *ex situ* populations of sable antelope relative to populations in the wild. Here we report the draft genome assembly of a male sable antelope, a member of the subfamily Hippotraginae (Bovidae, Cetartiodactyla, Mammalia). The 2.596 Gb draft genome consists of 136,528 contigs with an N50 of 45.5 Kbp and 16,927 scaffolds with an N50 of 4.59 Mbp. *De novo* annotation identified 18,828 protein-coding genes and repetitive sequences encompassing 46.97% of the genome. The discovery of single nucleotide variants (SNVs) was assisted by the re-sequencing of seven additional captive and wild individuals, representing two different subspecies, leading to the identification of 1,987,710 bi-allelic SNVs. Assembly of the mitochondrial genomes revealed that each individual was defined by a unique haplotype and these data were used to infer the mitochondrial gene tree relative to other hippotragine species. The sable antelope genome constitutes a valuable resource for assessing genome-wide diversity and evolutionary potential, thereby facilitating long-term conservation of this charismatic species.

The sable antelope (*Hippotragus niger*) is a large (>225 kg) ruminant endemic to the wooded savannahs of eastern and southern Africa. It is a member of the bovid subfamily Hippotraginae, which also includes the roan antelope (*H. equinus*), addax (*Addax nasomaculatus*), and four oryx (*Oryx*) species (Beisa oryx, *O. beisa*; scimitar-horned oryx, *O. dammah*; gemsbok, *O. gazella*; and Arabian oryx, *O. leucoryx*) as well as the extinct bluebuck (*H. leucophaeus*) (Bibi 2013; Robinson *et al.* 1996). At least four subspecies of sable antelope have been recognized based on morphological features and mitochondrial DNA sequence data (Ansell 1971; Matthee and Robinson 1999; Pitra *et al.* 2002; Pitra *et al.* 2006; Jansen van Vuuren *et al.* 2010; Rocha 2016; Vaz Pinto 2019): Zambian sable (*H. n. kirkii*); southern sable (*H. n. niger*); eastern sable (*H. n. roosevelti*); and giant sable (*H. n. variani*). The former three are listed as ‘Least Concern’ in the IUCN Red List of Threatened Species, whereas the giant sable antelope is categorized as ‘Critically Endangered’ and is listed on Appendix I of CITES (IUCN SSC Antelope Specialist Group 2008). A fifth genetic group, known as West Tanzanian sable, was recently defined based on its genetic divergence and discrete geographical distribution (Vaz Pinto 2019). In 1999, the world sable antelope population was estimated at 75,000 individuals, with 50% occurring in and around protected areas and 25% in *ex situ* collections (East 1998). Sable antelope, like many of the world’s largest herbivores with ≥100 kg body mass, face an increasing threat of extinction from habitat loss as well as hunting and poaching. Recent estimates show that the species has lost 51% of its former range, largely due to loss of woodland savannah from human population growth (Ripple *et al.* 2015).

Sable antelope were first imported into North America to the Smithsonian National Zoological Park (Washington, D.C.) in 1913 (Piltz *et al.* 2016). By 1991, the population had increased to 348 individuals in zoos accredited by the Association of Zoos and Aquariums (AZA), but has since declined to about 149 individuals (Piltz *et al.* 2016). Most of these comprise a Species Survival Plan (SSP) program, where the Sable Antelope Studbook is used to calculate mean kinships to guide best animal pairings. Estimates suggest that the current SSP population is descended from 39 founders. Almost all sable antelope that have been imported into North America originated from the southern sable subspecies (*H. n. niger*), although some Zambian sable (*H. n. kirkii*) were imported in 2000. Also of significance is the existence of more than 3,000 sable antelope maintained on private ranches in the USA, primarily in Texas (Mungall 2018). These animals are managed using less stringent (or no) genetic management practices, usually in herds with occasional bull rotations. Because relatedness among the original imported founders is unknown and early breeding records are scant or sporadic, the majority of the pedigree of sable antelopes managed by the SSP is unknown. Specifically, only 27% of the pedigree of animals included in the SSP Sable Antelope Studbook is known prior to assumed parental relationships and exclusions; with assumed parental relationships and exclusions, this value is 35% (Piltz *et al.* 2016). None of the animals in this population has ever been assessed using genetic approaches to obtain empirically-based estimates of genetic diversity, inbreeding status, or relatedness.

Our goal was to develop resources based on whole genome sequencing that will serve as a foundation for addressing questions related to the genetic status of the *ex situ* populations of sable antelope within North America relative to populations in the wild. We performed *de novo* sequencing of one individual to generate a draft quality assembly of the genome (*sensu* Mardis *et al.* 2002) followed by re-sequencing of seven additional individuals representing two subspecies. We provide an annotation of the species’ genome, including genes, repeat sequences, and single nucleotide variants (SNVs). We discuss how the genomic resources can be applied to conserving this charismatic antelope.

## Materials and Methods

### Sample collection and DNA preparation

**Figure 1:**
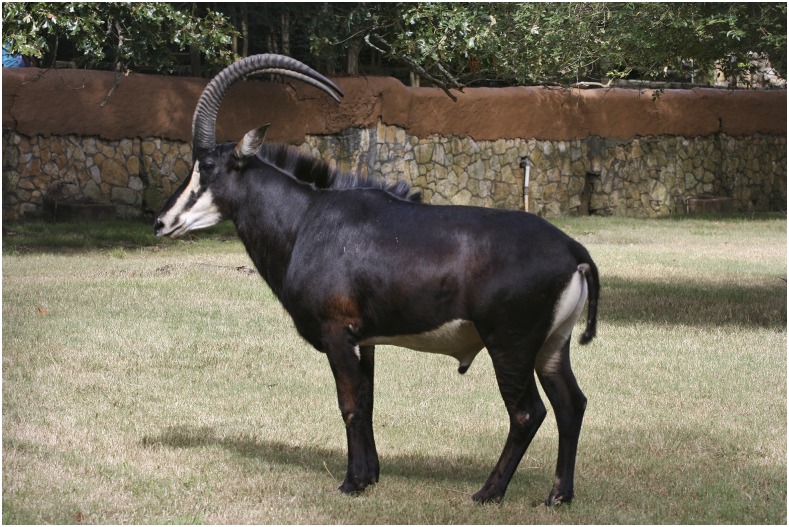
Photograph of SB#2152 at the Jackson Zoo, Jackson, Mississippi, USA. Photo credit: Dave Wetzel, Deputy Director, Jackson Zoo.

Whole blood or tissues were obtained from six sable antelope that originated from captive animals in the United States (Table 1). Five of these animals belonged to the southern sable antelope subspecies, *Hippotragus niger niger*: studbook [SB] #2152, SB#134, SB#381, SB#1954, SB#2130, and one belonged to the Zambian sable antelope subspecies, *H. n. kirkii*: SB#2027. Furthermore, one southern (HN250) and one Zambian (HN216) sable antelope were obtained from the wild to provide a comparison of genome-wide diversity with the individuals from zoos. For *de novo* sequencing and assembly of the reference genome, SB#2152, a male southern sable antelope maintained at the Jackson Zoo, Mississippi, was chosen from a pool of potential candidates (Figure 1). This individual was selected because its pedigree history included three confirmed events of consanguineous mating, with the expectation that genome-wide heterozygosity would be reduced and thereby facilitate *de novo* assembly. The coefficient of inbreeding (*F*) from the known pedigree of this individual (Figure S1), is *F* = 0.021.

**Table 1:** Metadata of sable antelope samples used for whole genome sequencing

Individual	Subspecies	Sex	Origin	History	Coverage	BioSample IDs
SB#2152	*Hippotragus niger niger*	Male	captive	Born 2003 at The Wilds, Cumberland, Ohio	40.56	SAMN07620900
SB#134	*Hippotragus niger niger*	Male	captive	Born 1970 at the San Francisco Zoo to wild caught parents from Zimbabwe	7.66	SAMN07620902
SB#1954	*Hippotragus niger niger*	Female	captive	Born at San Diego Safari Park	7.20	SAMN07620904
SB#2027	*Hippotragus niger kirkii*	Male	captive	Born at Glenwoods Farm, South Africa, imported into San Diego Safari Park	7.26	SAMN07620905
SB#2130	*Hippotragus niger niger*	Male	captive	Born 1999 at Safari Enterprises, Boerne, Texas	7.44	SAMN07620906
SB#381	*Hippotragus niger niger*	Male	captive	Born 1978 at Busch Gardens, Virginia	7.22	SAMN07620903
HN216	*Hippotragus niger kirkii*	Male	wild	Lusaka-Kafue region, Zambia	12.52	Available from the authors
HN250	*Hippotragus niger niger*	Female	wild	Mahango Game Reserve, Namibia	11.74	Available from the authors

Whole blood from SB#2152 was collected in a sterile Becton Dickinson Vacutainer vial and shipped on dry ice to the Smithsonian's National Zoological Park-Conservation Biology Institute, Washington, D.C. High molecular weight genomic DNA was extracted using the QIAamp DNA Blood Mini Kit (Qiagen, USA). Genomic DNA from SB#134, SB#381, SB#1954, SB#2130, and SB#2027 were obtained from tissues stored in the Frozen Zoo at the San Diego Zoo Institute for Conservation Research for re-sequencing. These DNAs were extracted using phenol-chloroform and purified using ethanol precipitation (modification of Sambrook *et al.* 1989) or with a QIAamp DNA kit (Qiagen, USA). All extracted DNA samples were checked and visualized on a 1.5% agarose gel run in 1x TBE buffer to ensure presence of high molecular weight DNA. DNA extracts were quantified using the Qubit 2.0 Fluorometer (Thermo Fisher Scientific, USA) following the manufacturer’s protocol. Genomic DNAs were converted into genomic library preparations and sequenced in a commercial facility (Macrogen Corp., Rockville, MD). All animal work was conducted in compliance with institutional rules and ethics.

### Ancestry assignment

The six sable antelope originating from zoos were scored for a set of 50 polymorphic microsatellites following Vaz Pinto *et al.* (2015) and Vaz Pinto (2019) to confirm population/subspecies assignment and to detect signals of possible admixture between subspecies. Amplifications were performed twice for each sample to exclude possible allele dropout errors, and PCR products were separated by size in an ABI3130xl Genetic Analyzer. Allele sizes were scored against the GeneScan 500 LIZ Size Standard, using GENEMAPPER 4.0 (Applied Biosystems). We used a Bayesian clustering analysis to assign the genotypes of the six individuals from zoos to five population groups known in Africa to ascertain their population of origin (Vaz Pinto 2019). This was performed using a reference dataset of 400 African wild sable antelope from Vaz Pinto (2019) that were previously genotyped for the same markers. The software STRUCTURE 2.3.4 (Falush *et al.* 2003) was run using the admixture model, correlated allele frequencies, and no prior geographical information. We performed 10 independent runs of 10^6^ MCMC sampling iterations following a burn-in period of 10^5^ steps, assuming K = 5, based on the findings of Vaz Pinto (2019) that wild sable antelope populations are structured into five genetic clusters. The 10 runs resulted in similar individual membership assignments.

### Sequencing

From the genomic DNA of sable antelope SB#2152, three paired-end libraries with a fragment size of 250 bp and one mate pair library with insert size of ∼5 Kb were prepared using the TruSeq DNA Sample Preparation Kit and the Nextera Mate Pair Library Preparation Kit, respectively, following the manufacturer’s instructions (Illumina, USA). For each library, paired-end sequencing was performed (2 × 101 bp) on a HiSeq 2000. For the five sable antelope provided by the San Diego Zoo Institute for Conservation Research and the two individuals from the wild, a paired-end library (200-500 bp) was constructed for each individual using the TruSeq DNA Sample Preparation Kit (Illumina, USA) and sequenced on a HiSeq2000 or HiSeq1500. Sequencing reads were processed using CASAVA v1.8.2 (Illumina, USA).

### Genome assembly

The pre-processed reads of sable antelope SB#2152 were first assembled *de novo* using ALLPATHS-LG with default settings (Gnerre *et al.* 2011), which resulted in an assembly that was quite fragmented: 403,030 contigs (N50 = 10,239 bp) and 71,644 scaffolds (N50 = 182,059 bp). To obtain an assembly with a higher contiguity, we used the MaSuRCA v3.2.8 assembler (Zimin *et al.* 2013). For Illumina-only assemblies, MaSuRCA follows a pipeline of error correction using QUORUM (Marçais *et al.* 2015) and then super-read construction by creating a k-mer look-up table using Jellyfish (Marçais and Kingsford 2011) and extending each k-mer that can be extended unambiguously (*i.e.*, of the possible k-mers with k-1 overlaps, only one exists in the lookup table) in both the 5′ and 3′ ends until there is no longer an unambiguous extension. Finally, this was followed by overlap, layout, and consensus (OLC) assembly and scaffolding of super-reads in a modified version of the CABOG assembler (Miller *et al.* 2008).

### Genome annotation and completeness

We used the RepeatMasker software (http://www.repeatmasker.org/) and the mammal-specific library from the Repbase Update library version 20170127 (Jurka 2000) to estimate the overall repeat content of the genome. RepeatMasker annotation included interspersed genomic repeats, tandem repeats identified using the Tandem RepeatFinder v4.09 software (Benson 1999), and low complexity sequences.

We used Augustus 3.2.3 (Stanke *et al.* 2008) to identify genes in the RepeatMasker-masked assembled sequence of the sable antelope genome. Augustus was launched with options –UTR = off, –softmasking = 1 and –species = human; these options disabled annotation of untranslated regions, interpreted the masked sequence as evidence against exons, and used the human gene models for gene prediction. Next, we filtered the obtained set of candidate genes by annotating their predicted proteins with InterProScan (Jones *et al.* 2014) and eggNOG-mapper (Huerta-Cepas *et al.* 2017) and removing genes for which proteins lacked annotated features. The annotation by eggNOG-mapper was based on eggNOG 4.5 orthology data (Huerta-Cepas *et al.* 2016).

We assessed the gene completeness of the SB#2152 assembly in Benchmarking Universal Single-Copy Orthologs (BUSCO) v3.0.2 (Waterhouse *et al.* 2018) using the Mammalia OrthoDB 9 BUSCO gene set (Zdobnov *et al.* 2017) and the long option (which performs species-specific gene model training). To further assess the quality of the assembly, we ran the QUAST v5.0.1 pipeline (Gurevich *et al.* 2013).

### Identification of single nucleotide variants

Single nucleotide variants (SNVs) were called from alignments of the re-sequenced reads to the assembled reference genome of SB#2152. The read alignment was performed using BWA 0.7.17 (Li and Durbin 2009). Bi-allelic SNVs were obtained from the alignments using a multistage variant filtering procedure that was implemented using the bcftools (Li 2011) and BEDtools (Quinlan and Hall 2010) packages and GNU Parallel (Tange 2018). SNVs were removed according to the following criteria: 1) all SNVs in the repeat-masked portion of the genome because SNV-calling in such regions is unreliable due to problems with short read alignment and assembly of repetitive elements (Reumers *et al.* 2011); 2) multiallelic SNVs; 3) SNVs having the alternative homozygous genotype for the reference individual; 4) SNVs with missing genotypes; 5) SNVs located within 10 base pairs of an indel; 6) SNVs with fewer than three reads supporting a genotype; and 7) SNVs with a variant quality score (*Q*) of less than 50. SNV effects with respect to the annotated protein-coding genes were predicted using SnpEff 4.3T (Cingolani *et al.* 2012).

### Mitochondrial genome assembly and analysis

Trimmed sequence reads from the eight individuals were mapped to the published mitochondrial genome of a sable antelope (GenBank accession JN632648; Hassanin *et al.* 2012) using Bowtie 2 v2.2.6 (Langmead and Salzberg 2012). SAMtools and BCFtools (Li *et al.* 2009) were used to generate a sorted BAM file as well as a .VCF file for the complete mitochondrial genome. A consensus FASTQ file was built using a minimum coverage of 100 reads. Seqtk (https://github.com/lh3/seqtk) was then used to convert the FASTQ file to a FASTA file.

The eight mitochondrial genomes were then combined into an alignment that also included whole mitochondrial genome sequences downloaded from GenBank of the following taxa: *Hippotragus niger variani* (KM245339), *Hippotragus niger* (JN632648), *Hippotragus equinus*, roan antelope (JN632647), *Addax nasomaculatus*, addax (JN632591), *Oryx beisa*, East African oryx (JN632676), *O. dammah*, scimitar-horned oryx (JN632677), *O. gazella*, gemsbok (JN632678), *O. leucoryx*, Arabian oryx (JN632679), *Alcelaphus buselaphus*, hartebeest (JN632593), and *Connochaetes taurinus*, blue wildebeest (JN632626). The alignment was estimated using the MAFFT v7.309 (Katoh and Standley 2013) plugin in Geneious R10.2.3 (https://www.geneious.com) with the following settings: Algorithm = Auto, scoring matrix = 200PAM/k = 2, gap open penalty = 1.53, offset value = 0.123. We then reconstructed a maximum likelihood phylogeny of these sequences using RAxML v8.0 (Stamatakis 2014) with the GTRGAMMA+P-Invar model of substitution and 500 bootstrap replicates, using the ML + thorough bootstrap tree search setting and branch lengths saved in the bootstrap trees (BS brL enabled).

### Data availability

The BioProject and BioSample accessions for the reference genome sequence and assembly of *Hippotragus niger* SB#2152 are PRJNA403773 and SAMN07620900, respectively. For the five whole genome re-sequenced individuals from the San Diego Zoo, the BioProject accession is PRJNA403774 and the BioSample accessions are SAMN07620902 (SB#134), SAMN07620903(SB#381), SAMN07620904 (SB#1954), SAMN07620905 (SB#2027), and SAMN07620906 (SB#2130). The assembled whole-genome sequence of SB#2152 has been submitted to the NCBI Genome database. The reads from the six sable antelope were also deposited in the SRA data repository (SRR8366604, SRR8366605, SRR8366606, SRR8366607, SRR8366677, SRR8366678, SRR8366679, SRR8366680, SRR8366681). Supplemental material available at FigShare: https://doi.org/10.25387/g3.7712603.

## Results and Discussion

### Ancestry assignment

We assessed the provenance of the six sable antelope originating from zoos by comparing them against a reference panel of 400 African wild sable antelope based on composite genotypes at 50 microsatellite loci. The average expected heterozygosity (*H_e_*) across the 50 loci was 0.500 for the southern sable antelope and 0.534 for Zambian sable antelope, as calculated in Arlequin v3.5.2.2 (Excoffier and Lischer 2010). The *H_e_* = 0.573 across the 50 loci for the five southern sable antelope that were whole genome sequenced. Individual membership assignment (*qi*) using a threshold of 0.85 revealed that SB#2027 shared a high degree of genetic ancestry with wild Zambian sable antelope (*qi* = 0.907) as expected, whereas three of the southern sable antelopes (SB#2152, SB#1954, SB#381) showed ancestry assignments consistent with wild counterparts of this subspecies (Table 2). Two of the southern sable antelopes (SB#2130, SB#134) demonstrated evidence of possible admixture with Zambian sable antelope.

**Table 2:** **Individual membership assignment (*qi*) of six captive sable antelopes from zoos in the USA to five clusters (K = 5) using wild African reference samples previously validated (****Vaz Pinto 2019****). All samples were genotyped for 50 microsatellites (see Methods). Bolded numbers refer to *qi* thresholds ≥0.85, indicating shared genetic ancestry and assignment to that genetic cluster or population. Missing data indicates the number of microsatellite loci out 50 for which genotype could not be generated for a particular sample.**

Sample	Missing loci	Eastern	Western Tanzania	Zambian	Angolan	Southern
**SB#134**	3	0.020	0.042	0.152	0.015	0.771
**SB#381**	2	0.043	0.050	0.029	0.014	**0.863**
**SB#1954**	0	0.009	0.023	0.060	0.009	**0.900**
**SB#2027**	0	0.011	0.016	**0.907**	0.021	0.045
**SB#2130**	1	0.010	0.013	0.234	0.025	0.718
**SB#2152**	2	0.027	0.029	0.033	0.010	**0.900**

### Genome assembly

Sequencing of the three joined paired-end and the mate pair libraries of SB#2152 generated 1,164,754,760 reads (117,640,230,760 bp) and 438,317,014 reads (44,270,018,414 bp), respectively (Table S1). Across the four libraries sequenced for SB#2152, total and effective (*i.e.*, the number of reads retained after filtering) sequence coverage was 45x and 40.5x, respectively. The number of total bases generated for the seven re-sequenced individuals ranged from 19,995,630,540 to 35,471,415,924 bp (197,976,540 to 281,519,174 reads). Q20 base scores were >93% for all animals. For the seven re-sequenced individuals, coverage ranged from ∼7x to 12.5x.

The SB#2152 draft assembly generated using MaSuRCA v3.2.8 contained 136,528 contigs (2,562,010,215 bp) with an N50 of 45,499 bp that were then assembled into 16,927 scaffolds (2,595,530,148 bp) with an N50 of 4.59 Mbp (Table 3). BUSCO evaluation of gene completeness showed that 3,890 out of 4,104 genes (94.8%) were complete, and only 113 genes (2.7%) were found missing (Table 3). The estimated genome size was 2.926 Gb based on an analysis of *k*-mer frequency (Marçais and Kingsford 2011), which is comparable to the genome sizes of the domestic cow (2.92 Gb) and the gemsbok (3.2 Gb), another member of the Hippotraginae (Zimin *et al.* 2009; Farré *et al.* 2019).

**Table 3:** Whole genome assembly statistics and BUSCOv3 scores based on the MaSuRCA v3.2.8 assembly of the SB#2152 sable antelope

QUAST results		
Statistic	Contig (bp)	Scaffold (bp)
**N10**	116,388	12,177,738
**N20**	86,857	8,975,322
**N30**	69,004	7,052,697
**N40**	56,230	5,820,171
**N50**	45,500	4,586,323
**L10**	1,708	18
**L20**	4,283	43
**L30**	7,601	76
**L40**	11,731	116
**L50**	16,801	167
**Longest segment**	399,521	19,097,140
**Total length**	2,562,048,600	2,595,532,220
**Total number**	136,532	16,931
**% GC content**	41.79	41.25
BUSCOv3 results		
Category	Total number	Percentage
**Complete BUSCOs**	3,890	94.8%
**Complete and single-copy BUSCOs**	3,845	93.7%
**Complete and duplicated BUSCOs**	45	1.1%
**Fragmented BUSCOs**	101	2.5%
**Missing BUSCOs**	113	2.7%
**Total number BUSCO groups**	4,104	—

### Annotation

The estimated GC content of the SB#2152 genome using contigs was 41.8%, similar to the G+C content of other mammalian genomes (*e.g.*, cow = 41.7%; human = 40.8%) (Zimin *et al.* 2009; Lander *et al.* 2001). *De novo* prediction using Augustus 3.2.3 and human gene models resulted in a set of 21,276 candidate protein-coding genes in the sable antelope reference assembly. This quantity is comparable to the 20,892 and 21,426 protein-coding genes found in the domestic cow and Tibetan antelope genomes, respectively, but lower than the 23,125 reference gene set in the gemsbok (Zimin *et al.* 2009; Ge *et al.* 2013; Farré *et al.* 2019). The candidate gene set was then filtered using eggNOG 4.5 orthology data (Huerta-Cepas *et al.* 2016), which reduced the set to 18,828 protein-coding genes.

An estimated 46.97% (1,219,061,301 bp) of the genome was composed of repetitive sequence, based on masking of non-long terminal repeat (LTR) retrotransposons (SINEs and LINEs), LTR elements, DNA elements, small RNAs, low complexity sequences, and simple and complex tandem repeats (Table 4). This percentage of repetitive element content was similar to the domestic cow (45.28%) and European bison (47.3%) but higher than in the Tibetan antelope (36.72%) (Zimin *et al.* 2009; Wang *et al.* 2017; Ge *et al.* 2013). Among repetitive sequences within transposable elements, 11.4% were represented by SINEs and 25.54% by LINEs. The percentage of the latter class of transposable elements is highly consistent with that observed in the gemsbok assembly (Farré *et al.* 2019). There were fewer SINEs than reported in Tibetan antelope (15.41%) and cow genomes (16.26%), whereas the number of LINEs was higher compared to the Tibetan antelope genome (16.12%). Long terminal repeat elements accounted for 5.15% of repetitive sequences, comparable to that found in the cow (4.46%) and Tibetan antelope (3.81%) genomes. BovB-LINE1 constituted a major fraction of the LINE retrotransposons, consistent with the expansion of these elements during the evolution of the Bovidae (Szemraj *et al.* 1995; Adelson *et al.* 2009; Nilsson *et al.* 2012). We also found that approximately 536 Mb of the genome was composed of an 804 bp bovine-specific satellite DNA, which is usually located in the centromeric and pericentric regions of chromosomes (D’Aiuto *et al.* 1997; Kopecna *et al.* 2014).

**Table 4:** Summary of repetitive element content found in the SB#2152 sable antelope genome assembly

	Number	Length occupied (bp)	Percent masked
**SINEs**	2,170,055	295,983,485	11.40%
**LINEs**	1,396,799	662,785,934	25.54%
**LTR elements**	430,413	133,548,072	5.15%
**DNA elements**	310,575	62,258,735	2.40%
**Unclassified**	4,324	771,329	0.03%
**Total interspersed repeats**	—	1,155,347,555	44.52%
**Small RNA**	252,281	42,527,077	1.64%
**Satellites**	93,024	40,978,135	1.58%
**Simple repeats**	462,487	18,805,146	0.72%
**Low complexity**	75,644	3,668,178	0.14%

### Genome diversity

**Figure 2:**
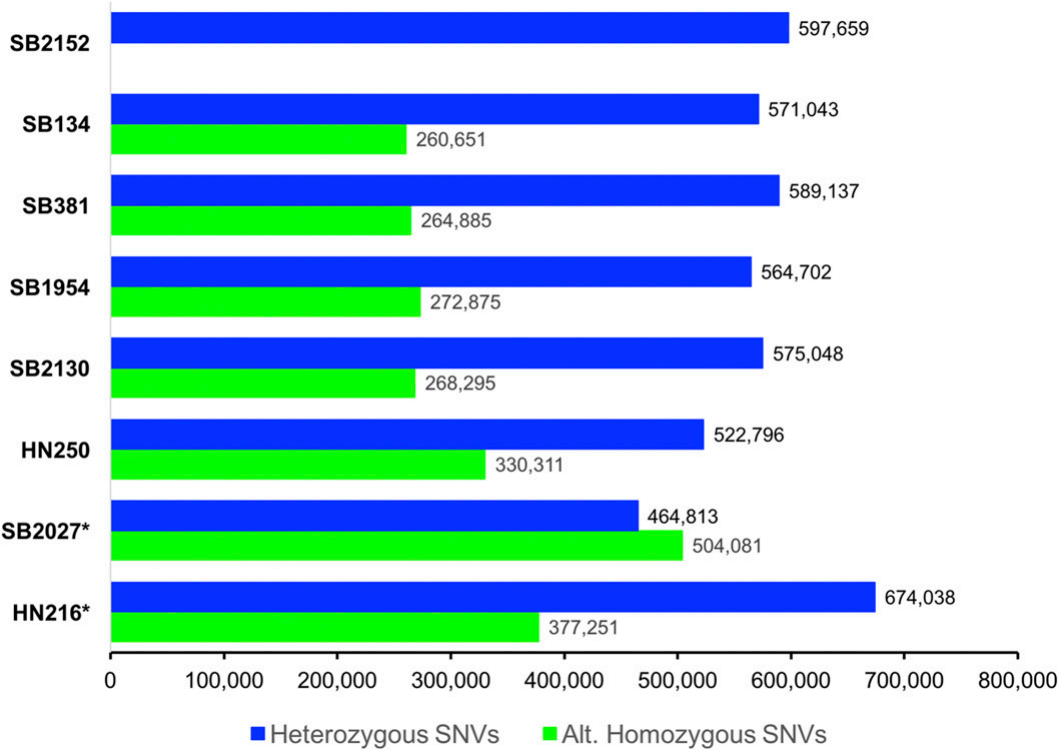
Bar chart comparing the number of high-quality (after filtering) heterozygous and alternative homozygous SNVs among the eight sable antelopes sequenced for this study. Note the relatively higher number of alternative homozygous SNVs in SB2027* and HN216*, which represent Zambian sable antelope (*H. n. kirkii*) whereas the other individuals represent southern sable antelope (*H. n. niger*).

We mapped the sequence reads of the seven sable antelope that were re-sequenced to the SB#2152 reference genome and identified a total of 15,405,064 SNVs. These SNVs were then filtered according to a multistage filtering approach based on several criteria (Table S2), resulting in a final set of 1,987,710 bi-allelic SNVs across the eight sable antelope. The number of heterozygous SNVs in the six sable antelope originating from zoos ranged from 464,813 (SB#2027) to 597,659 (SB#2152). For the two individuals from the wild, HN216 and HN250, 674,038 and 522,796 heterozygous SNVs were observed, respectively. The number of homozygous SNVs in the seven re-sequenced individuals, where the SNV is fixed relative to the reference individual (SN#2152) ranged from 260,651 to 377,251. Interestingly, the two Zambian sable antelope (SB#2027 and HN216) showed a higher number of alternative homozygous SNVs relative to the six southern sable individuals (Figure 2), likely reflecting the population divergence between the two subspecies. Additionally, the wild sable HN250 exhibits the highest number of alternative homozygous SNVs among southern sables, a possible indication of the closed management of the *ex situ* sable population maintained in the USA. Analyses of the effects of SNVs with respect to annotated protein-coding genes using SnpEff identified 743,675 effects, of which 720,709 were located within introns. Of the 22,966 SNVs situated within exons, 11,350 were synonymous, 11,386 were missense SNVs, and 230 were identified as nonsense SNVs (29 variants losing a start codon and 201 variants gaining a stop codon). The overall transition/transversion ratio across SNVs was 2.14 (1,354,290/633,420).

**Figure 3:**
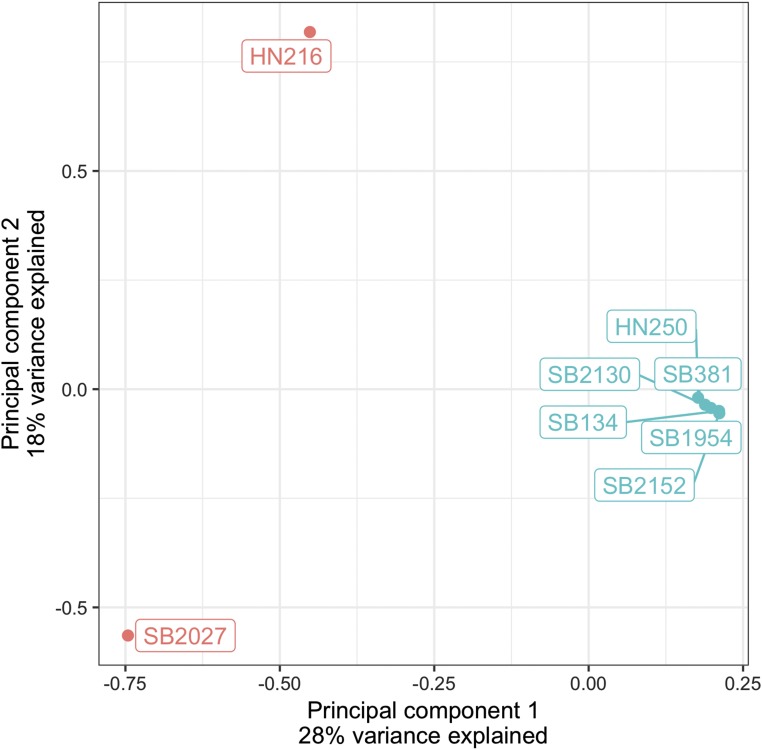
Plot of principal component analysis for the six southern sable antelope (*Hippotragus niger niger*, green dots) and two Zambian sable antelope (*Hippotragus niger kirkii*, red dots).

Principal component analysis of the eight sable antelope using the set of filtered bi-allelic SNVs revealed that the six individuals representing the southern sable antelope subspecies (*Hippotragus niger niger*) formed a cluster that was distinct from the two individuals representing the Zambian sable antelope subspecies (*H. n. kirkii*) (Figure 3). This axis (PC1) explains 28% of the variance. However, the two Zambian sable antelope, one from a zoo (SB#2027) and one from the wild (HN216), were not clustered together. Although these patterns are based on only a few individuals, our results are consistent with recent analyses of whole mitochondrial genomes from sable antelope populations across their remaining native range in Africa that show deep genetic divisions between both traditionally recognized subspecies and within subspecies, including *H. n. niger* and *H. n. kirkii* (Rocha 2016). An implication of these findings is that genome-wide SNVs can be used to trace the original source populations of captive animals as well as detect possible admixture and introgression between genetically distinct sable antelope populations.

### Mitochondrial genome and phylogeny

Assembly of the mitochondrial genome from the eight individuals resulted in a consensus sequence of 16,533 bp, slightly longer in length compared to the first mitochondrial genome published for this species (16,507 bp, Hassanin *et al.* 2012) or the one obtained from a giant sable antelope (16,504 bp, Themudo *et al.* 2015). Each of the eight sable antelopes defined a unique haplotype that differed by 11 to 87 substitutions (Kimura 2-parameter distances: 0.067–0.529%) and that also differed from the two previously published mitochondrial genome sequences (1-100 substitutions, 0.006–0.622%).

**Figure 4:**
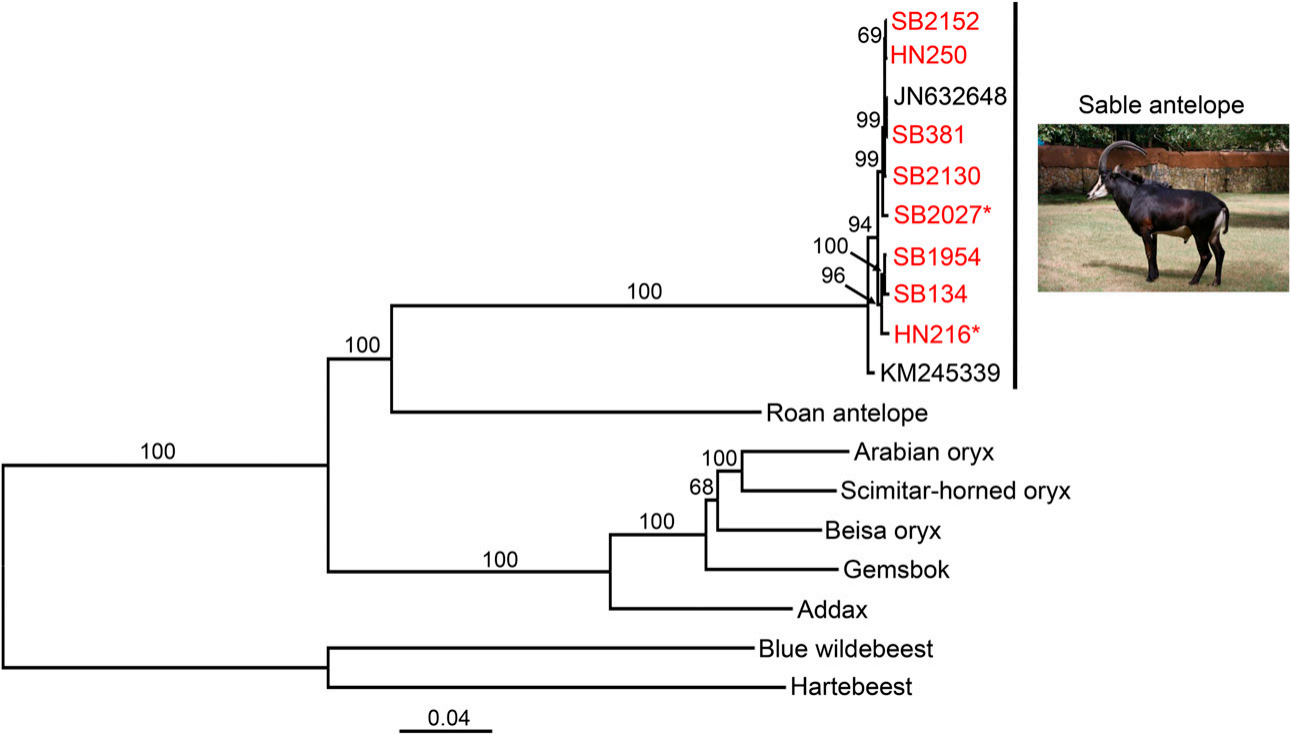
Maximum likelihood gene tree based on analysis of the mitochondrial genome showing the position of the eight sable antelopes sequenced (red font) in relationship to two previously reported sable antelope sequences and other species of the Hippotraginae. Asterisks indicate the two Zambian sable antelope individuals. Numbers shown above branches are bootstrap pseudo-replicates (out of 500). Branch lengths are proportional to the number of substitutions per site (scale bar). The tree is rooted with the blue wildebeest and hartebeest.

Phylogenetic analysis of the mitochondrial genomes (excluding the control region) using a maximum likelihood approach revealed that the 10 sable antelope sequences (eight from this study plus two from previous studies) clustered together with 100% bootstrap support, with the sequence of the giant sable antelope (*Hippotragus niger variani*, KM245339) falling outside the other sequences (Figure 4). We also note that the two Zambian sables, SB#2027 and HN216, fall into separate clades, consistent with the results of the principal component analyses and the strong mitochondrial genetic structure associated this population (Rocha 2016). The sable antelope sequences were sister to the roan antelope sequence that, in turn, grouped with the remaining species that constituted the Hippotraginae, with the branching order largely conforming to the topology found in comprehensive phylogenetic analyses of the Cetartiodactyla (Hassanin *et al.* 2012) or Ruminantia (Bibi 2013). Our topology is congruent with the topology found in a more focused study of the Hippotraginae, which also showed that the extinct blue antelope (*Hippotragus leucophaeus*) that was endemic to the coastal plains and highlands of southern Africa was the sister group of sable antelope (Themudo and Campos 2018).

## Conclusions

Our draft genome of the sable antelope represents an advance in the comparative genomics of the Bovidae. Following the sequencing and assembly of the gemsbok genome (Farré *et al.* 2019), it is the second genome sequenced from a member of the Hippotraginae, which has its roots in the early Miocene of Eurasia (Turner and Anton 2004; Solounias 2007). We generated an initial annotation of protein-coding genes and repetitive sequence content, and characterized SNV diversity across autosomal regions and the mitochondrial genome among six individuals from zoos and two individuals from the wild, representing at least two of the known subspecies or genetic lineages (Ansell 1971; Vaz Pinto 2019). The genomic data we have generated provides an important foundation for understanding and monitoring genome-wide diversity that is fundamental to managing populations to achieve sustainability, including clarifying founder animals, identifying genetically valuable, but under-represented individuals, improving breeding recommendations, and recognizing admixture that could compromise species integrity. Identification of hundreds of thousands of high-quality SNVs provides an important resource for studying genome-wide diversity, inbreeding status, admixture, and demographic processes in both *in situ* and *ex situ* populations of sable antelope. Our draft assembly of the sable antelope genome serves as a foundation for a chromosomal-level reference genome that can be generated with the addition of chromosome conformation data such as Hi-C contact maps (Dudchenko *et al.* 2018).
